# Efficacy of adjuvant chemotherapy for non-small cell lung cancer assessed by metastatic potential associated with ACTN4

**DOI:** 10.18632/oncotarget.8890

**Published:** 2016-04-21

**Authors:** Nami Miura, Masahiro Kamita, Takanori Kakuya, Yutaka Fujiwara, Koji Tsuta, Hideaki Shiraishi, Fumitaka Takeshita, Takahiro Ochiya, Hirokazu Shoji, Wilber Huang, Yuichiro Ohe, Tesshi Yamada, Kazufumi Honda

**Affiliations:** ^1^ Division of Chemotherapy and Clinical Research, National Cancer Center Research Institute, Tokyo 104-0045, Japan; ^2^ Department of Thoracic Oncology, National Cancer Center Hospital, Tokyo 104-0045, Japan; ^3^ Division of Pathology, National Cancer Center Hospital, Tokyo 104-0045, Japan; ^4^ Department of Functional Analysis, Fundamental Innovative Oncology Core Center, National Cancer Center Research Institute, Tokyo 104-0045, Japan; ^5^ Division of Molecular and Cellular Medicine, National Cancer Center Research Institute, Tokyo 104-0045, Japan; ^6^ Abnova, Taipei 114, Taiwan; ^7^ Japan Agency for Medical Research and Development: AMED-CREST, AMED, Tokyo 100-0004, Japan

**Keywords:** ACTN4 (actinin-4), predictive biomarker for adjuvant chemotherapy, early stage of non-small cell lung cancer (NSCLC), JBR.10

## Abstract

Although several clinical trials have demonstrated the benefits of platinum-combined adjuvant chemotherapy for resected non-small cell lung cancer (NSCLC), predictive biomarkers for the efficacy of such therapy have not yet been identified. Selection of patients with high metastatic ability in the early stage of non-small cell lung cancer (NSCLC) has the potential to predict clinical benefit of adjuvant chemotherapy (ADJ).

In order to develop a predictive biomarker for efficacy of ADJ, we reanalyzed patient data using a public database enrolled by JBR.10, which was a clinical trial to probe the clinical benefits of ADJ in stage-IB/II patients with NSCLC. The patients who were enrolled by JBR.10 were classified into 2 subgroups according to expression of the ACTN4 transcript: ACTN4 positive (ACTN4 (+)) and ACTN4 negative (ACTN4 (−)). In the ACTN4 (+) group, overall survival (OS) was significantly higher in the ADJ subgroup compared with the observation subgroup (OBS), indicating a significant survival benefit of ADJ. However, no difference in OS was found between ADJ and OBS groups in ACTN4 (−). Although ACTN4 expression level did not correlate with the chemosensitivity of cancer cell lines for cytotoxic drugs, the metastatic potential of A549 lung adenocarcinoma cells was significantly reduced by ACTN4 shRNA in in vitro assays and in an animal transplantation model. The clinical and preclinical data suggested that ACTN4 is a potential predictive biomarker for efficacy of ADJ in stage-IB/II patients with NSCLC, by reflecting the metastatic potential of tumor cells.

## INTRODUCTION

Recent clinical trials have led to the adoption of adjuvant cisplatin-based chemotherapy for patients with resected stages-IB to IIIA NSCLC [1–4]. However, the increased effect on overall survival (OS) of adjuvant cisplatin-based chemotherapy is not always sufficient. In particular, no clinical trial has shown a substantial survival benefit in stage-IB. To establish a predictive biomarker for selection of patients for which adjuvant cisplatin-based chemotherapy will have efficacy, Zhu et al. identified a gene signature that can determine patients for whom adjuvant chemotherapy (ADJ) with cisplatin and vinorelbin will be effective [5]. This gene signature was obtained from analysis of gene expression profiles of samples of patients with stage-IB and -II who were enrolled in JBR.10, which is a phase-III randomized trial of ADJ with cisplatin and vinorelbin versus an observation group in completely resected stage-IB, -II and IIIA NSCLC [4, 6]. The database in which these gene expression profiles and clinical information of individual patients and survival time were described is open to the public on the internet (http://www.ncbi.nlm.nih.gov/geo/query/acc.cgi?acc=gse14814) [5].

If it is considered that the survival benefit of ADJ is due to the successful control of micro metastatic lesions; therefore, a biomarker that can predict the existence of micro metastasis that cannot be detected by imaging modality would be important for the decisions of therapeutic strategy for ADJ in patients with stage-IB and -II NSCLC.

Actinin-4 (ACTN4) is an actin-bundling protein that we identified in 1998 [7]. We demonstrated that overexpression of ACTN4 leads to an aggressively malignant phenotype of cancer cells with metastatic potential [7–9]. We recently demonstrated that patients with gene amplification of ACTN4 in stage-I lung adenocarcinoma who never underwent ADJ with any drug definitely have a worse prognosis than patients without gene amplification of ACTN4, and we reported the potential clinical applicability of ACTN4 as a prognostic biomarker of stage-I adenocarcinoma [10].

In this study, we investigated the clinical applicability of ACTN4 as a predictive biomarker for survival benefit of ADJ.

## RESULTS

### Reanalysis of the survival benefit of ADJ in subgroups of ACTN4 expression in patients enrolled in JBR.10

**Figure 1:**
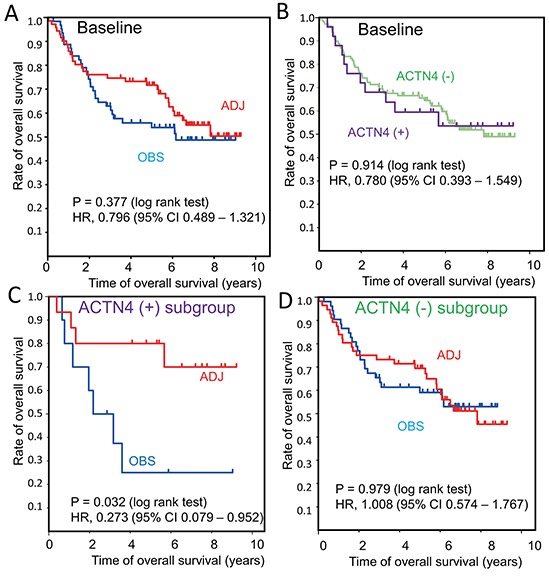
Overall survival curves in a reanalysis of a public database of patient information enrolled in JBR.10 Kaplan Meier curves of overall survival were constructed for the following groups of patients: **A.** Baseline patients who underwent adjuvant chemotherapy (ADJ, red line) or who were observed without ADJ (OBS, blue line). **B.** Subgroup analysis of actinin-4 (ACTN4) (−) (green line) and ACTN4 (+) (purple line) in baseline patients. **C.** ADJ (red line) and OBS (blue line) in the ACTN4 (+) subgroup. **D.** ADJ (red line) and OBS (blue line) in theACTN4 (−) subgroup. Statistical significances for overall survival were calculated using the log rank test. Hazard ratios for ADJ compared with OBS were calculated by univariate Cox regression analysis.

The clinical information and mRNA profiles of 133 patients of stage-I/II who were enrolled by JBR.10 are described in the public database (http://www.ncbi.nlm.nih.gov/geo/query/acc.cgi?acc=gse14814). This patient group was comprised of a group of 71 cases who underwent ADJ and a group of 62 cases who were observed without ADJ (OBS) (Table 1). Significant clinical benefit of ADJ for overall survival time compared with OBS was not seen in the baseline data of 133 patients (log rank test; *P* = 0.377; hazard ratio (HR), 0.796; 95% confidence interval (95% CI), 0.489 – 1.321) (Figure 1A and Table 2). We then divided the patients into subgroups based on the presence or absence of ACTN4 expression, after defining a cut-off value for ACTN4 according to X-tile algorithms as the value that gives the lowest *P*-value in the OBS subgroup (Supplementary Figure S1) [11]. The cut-off value of ACTN4 was defined as 831.15 of the signal means of ACTN4 in the database that was analyzed by JBR.10. The 133 stage-I/II patients who were enrolled in this database were then classified into two subgroups based on this cut-off value of 831.15: an ACTN4 overexpression subgroup, ACTN4 (+) (*N* = 25) and a subgroup without ACTN4 overexpression, ACTN4 (−) (*N* = 108) (Table 1). Although there were no statistically significant differences between patient subgroups of ACTN4 (+) and ACTN4 (−) in terms of age, gender, clinical stage, or treatment after surgery, there was a statistically significant difference in pathological subtypes (p=0.018, Fisher's exact test). The overall survival times were not significantly different between ACTN4 (+) and ACTN (−) in the baseline data of these 133 patients (*P* = 0.914) (Figure 1B), which consisted of both ADJ and OBS subgroups. Within the ACTN4 (+) subgroup, the overall survival time of the ADJ group (*N* = 15) was significantly longer than that of the OBS group (*N* = 10) (*P* = 0.032) (Figure 1C). However, within the ACTN4 (−) subgroup, no statistically significant difference in overall survival time between the patients who underwent ADJ (*N* = 56) and the patients in the OBS group was found (*N* = 52) (Figure 1D). In the ACTN4 (+) subgroup, the hazard ratio (HR) for death of the patients treated with ADJ was significantly decreased in comparison with patients of the OBS group (HR 0.273, 95% confidence interval (95% CI) 0.079 – 0.952, *P* = 0.042) in both univariate and multivariate analysis (Table 3). In contrast, in the ACTN4 (−) subgroup, no statistically significant difference in the reduction of HR for death was seen between the OBS and the ADJ groups (HR 1.008, 95% CI 0.574 – 1.767, *P* = 0.979) (Table 4). These data suggested that overexpression of ACTN4 is a potential predictive biomarker for ADJ.

**Table 1:** Baseline demographics of JBR.10 patient subgroups with or without overexpression of ACTN4

	Total number *N* = 133)	ACTN4 (+)(*N* = 25)		ACTN4 (−) (*N* =108)		*P*-value
Average age (yr), +/− SD		63.18	7.98	60.26	9.22	0.12*
Sex						
Male	91	20		71		0.23**
Female	42	5		37		
Stage						
IB	73	16		57		0.38**
II	60	9		51		
Pathological subtype						
SCC^a^	52	15		37		0.018**
Non-SCC	81	10		71		
Treatment						
ADJ^b^	71	15		56		0.51**
OBS^c^	62	10		52		

* *P*-values, Student's *t*-test

** *P*-values, Fisher's exact test

a SCC: squamouse cell carcinoma

b ADJ; adjuvant chemotherapy

c OBS; observation

**Table 2:** Hazard ratio for death of patients in baseline of JBR.10

	Univariate analysis			Multivariate analysis		
	HR	95 % CI	p-value	HR	95% CI	p-value
**Age**						
<65/≥65	1.839	1.101-3.071	**0.0198**	1.89	1.131-3.157	**0.0151**
Sex						
Female/male	0.617	0.339-1.236	0.1145			
Stage of disease						
IB/II	1.734	1.042-2.884	**0.0341**	1.782	1.070-2.967	**0.0263**
Pahological subtype						
Non-SCC/SCC	0.620	0.359-1.069	0.0856			
Expression of ACTN4						
Positive/negative	1.036	0.588-1.994	0.9143			
Treatment recived						
Observation alone / adjuvant chemotherapy	0.796	0.479-1.321	0.3771			

The clinical data was according to refernce **. HR; hazard ratio, CI, confidatial interval, SCC: squamouse cell carcinoma.

**Table 3:** Hazard ratio for death of patients in the ACTN4 (+) subgroup

	Univariate analysis			Multivariate analysis		
	HR	95% CI	*P*-value	HR	95% CI	*P*-value
Age (yr)						
<65 / ≥65	1.786	0.544-5.862	*0.339*			
Sex						
Female / male	2.2	0.580-8.336	*0.246*			
Stage						
IB / II*	1.545	0.470-5.082	*0.474*			
Pahological subtype						
Non-SCC^a^/SCC	0.350	0.104-1.178	*0.090*			
Treatment*						
OBS^b^/ADJ^c^	**0.273**	**0.079-0.952**	***0.042***	**0.273**	**0.079-0.952**	***0.042***

HR; hazard ratio, 95% CI; confidence interval

* according to JBR.10

a SCC: squamouse cell carcinoma

b OBS; observation alone

c ADJ; adjuvant chemotherapy and the data in bold are statistically significant.

**Table 4:** Hazard ratio for death of patients in the ACTN4 (−) subgroup

	Univariate analysis			Multivariate analysis		
	HR	95% CI	*P*-value	HR	95% CI	*P*-value
Age (yr)						
<65 / ≥65	**1.857**	**0.047-3.293**	***0.0343***	1.639	0.917-2.929	0.096
Sex						
Female / male	**2.035**	**1.035-3.983**	***0.0382***	1.773	0.896-3.511	*0.1001*
Stage						
IB / II*	1.87	1.031-3.202	0.039	1.648	0.930-2.919	*0.087*
Pahological subtype						
Non-SCC^a^/SCC	0.691	0.371-1.284	*0.243*			
Treatment *						
OBS^b^/ADJ^c^	1.008	0.574-1.767	*0.979*			

HR; hazard ratio, 95% CI; confidence interval

* according to JBR.10

a SCC: squamouse cell carcinoma

b OBS; observation alone

c ADJ; adjuvant chemotherapy and the data in bold are statistically significant

### Evaluation of the involvement of ACTN4 in cell metastatic ability and other biological functions *in vitro*

**Figure 2:**
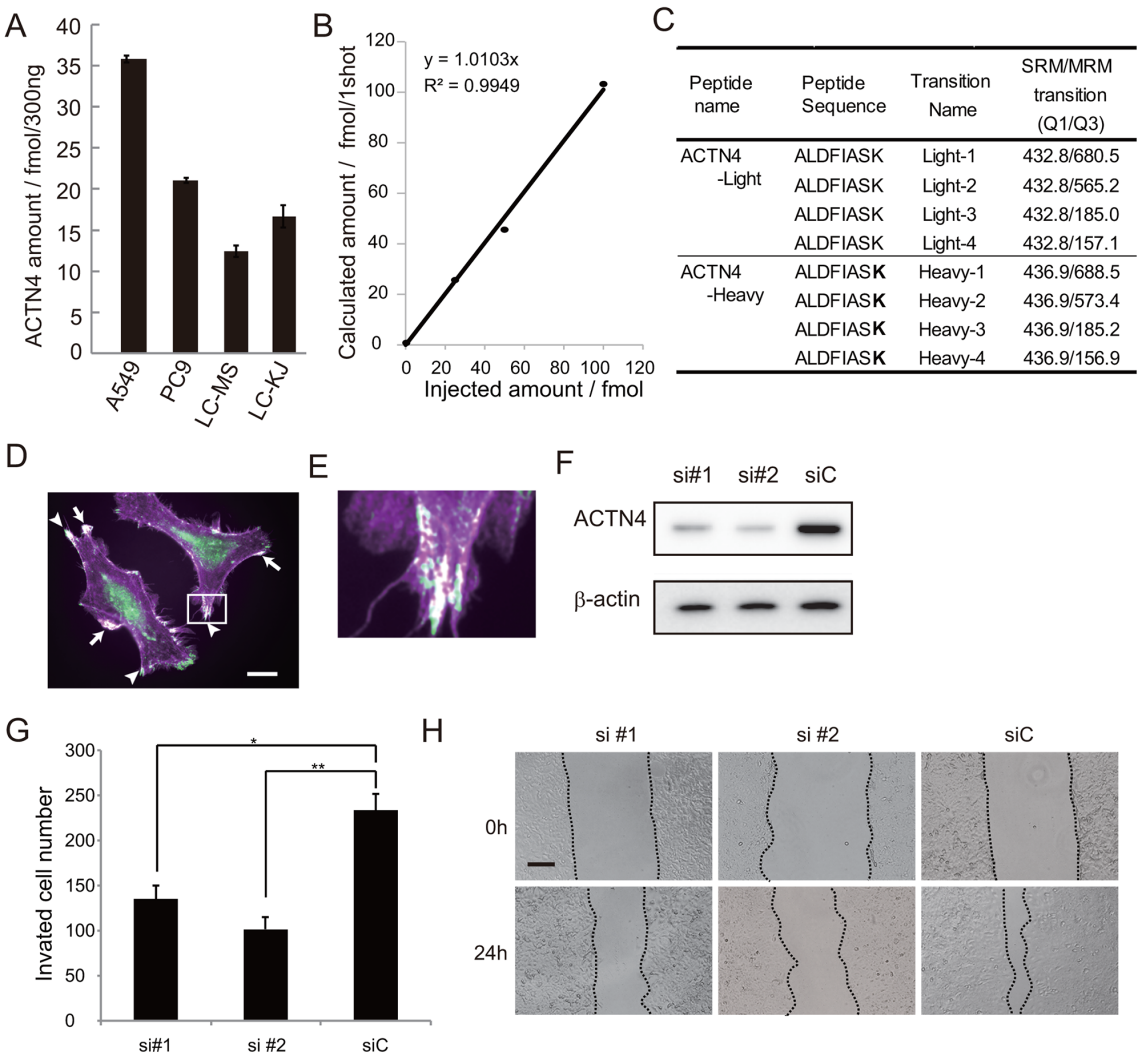
Effect of ACTN4 knockdown using siRNA of ACTN4 on in vitro cell migration ability **A.** Absolute amount of ACTN4 protein in non-small cell lung cancer (NSCLC) cell lines by multiple reaction monitoring (MRM) using mass spectrometry (MS). **B.** Standard curve of MRM with ACTN4 peptide. **C.** Peptide sequence and MRM transition for actinin-4 quantitation using MS. Bold letters indicate amino acid residues labeled with stable isotope (13C and 15N). **D, E.** Immunocytochemical analysis of ACTN4 (green) and phalloidin (violet) staining of A549 cells. Arrows indicate co-stained lamellipodia, and arrowheads indicate co-stained filopodia. Bar in D indicates 10 μm. **F.** Western blot analysis of ACTN4 expression in A549 cells transfected with ACTN4 (si#1 or si#2) or control (siC) siRNA. **G.** Boyden chamber assay of the effect of ACTN4 siRNAs on cell invasion. *P < 0.05, **P < 0.01 (*t*-test). **H.** Representative photographs of wound healing by cells transfected with siRNA of ACTN4 or control siRNA. The cells are shown at wounding (0 h) and 24 hours later (24 h). Bar indicates 100 μm.

**Figure 3:**
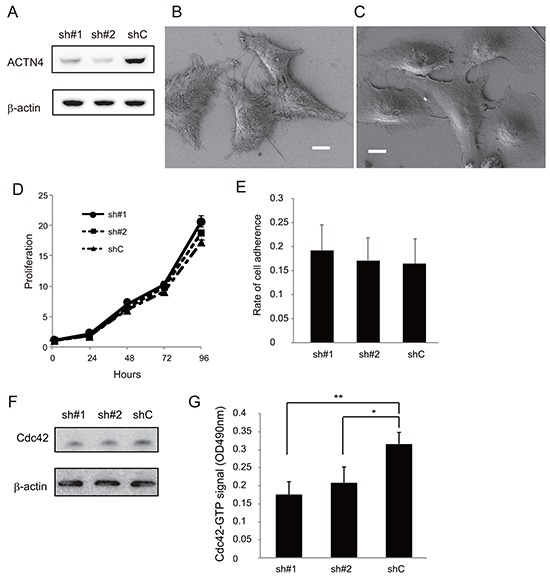
Effect of ACTN4 knockdown using shRNA of ACTN4 on in vitro proliferation, cdc42 activation and adherence to HUVEC **A.** Western blot analysis of ACTN4 expression in A549 cells infected with ACTN4 (sh#1 or sh#2) or control (shC) shRNA. **B, C.** Representative photographs of scanning electron microscopic analysis of A549 cells that were infected with control shRNA (B) or ACTN4 (C). Bars, 10 μm. **D.** Proliferation of the shRNA-infected A549 cells. **E.** Adherence of the A549 cells to HUVEC. **F, G.** Activation assay of Cdc42 in A549 cells infected with ACTN4 or control shRNAs. (F) Western blot analysis of Cdc42 in the total lysate of A549 cells. Beta-actin was blotted as a loading control. (G) Signal intensity of the active, Cdc42-GTP fraction in the A549 cells.

It is possible that ADJ is more effective for patients with high metastatic potential than for those without metastatic potential; the reason being that, if the patients undergo complete resection including micro metastatic lesions, ADJ is not needed for such patients whereas, in contrast, ADJ may be beneficial for patients who potentially have metastatic lesions beyond the limits of the resected area. Based on this hypothesis, we examined whether expression of ACTN4 could be used to evaluate the metastatic potential of lung cancer cell lines in an in vitro assay. Firstly, we absolutely quantified the expression level of the ACTN4 protein in these cell lines by multiple reaction monitoring (MRM) using mass spectrometry. The A549 cell line, which is an NSCLC cell line that was derived from patients with lung adenocarcinoma, has gene amplification of ACTN4 [12]. A549 showed the highest expression of the ACTN4 protein compared to the other lung cancer cell lines tested (Figure 2A–2C). Therefore, in order to examine the effect of gene amplification of ACTN4 on the biological behavior of NSCLC, we used A549 cells for further experiments. Immunofluorescence analysis indicated that the ACTN4 protein was mainly present in cellular processes that are considered to be associated with cell migration (Figure 2D and 2E). Transient transfection of A549 cells with small interfering RNAs (siRNAs) of ACTN4 reduced the level of expression of the ACTN4 protein (Figure 2F) and significantly decreased cell migration ability, as assessed using a Boyden chamber assay with Matrigel (Figure 2G) and in a wound healing assay (Figure 2H). Next, we engineered cell lines termed A549-luc-C8-KD-ACTN4s (sh#1 and sh#2), which expressed luciferase (luc), and in which ACTN4 was stably knocked down by infection with a lentivirus-based small hairpin RNA (shRNA). An A549-luc-C8-cont control cell line (shC) with control transduced particles was also engineered (Figure 3A). Consistent with the observed ACTN4 expression in cellular processes (Figure 2D and 2E), scanning electron microscopy showed that cellular processes that are considered to be associated with cell migration were significantly reduced by shRNAs of ACTN4 in comparison with control cells (Figure 3B and 3C). No statistically significant differences in cell proliferation were determined between A549-luc-C8-KD-ACTN4s and A549-luc-C8-cont (shC, sh#1, and sh#2) cells (Figure 3D). We also tested the potential ability of ACTN4 to modulate cell adhesion to endothelial cells. The ability of the cell lines to adhere to HUVEC endothelial cells was examined in a cell attachment assay. No statistically significant difference in adhesion to endothelial cells between A549-luc-C8-KD-ACTN4s and A549-luc-C8-cont cells was seen (Figure 3E). It is well known that the activation of cdc42 significantly stimulates the formation of filopodia, which are involved in cell migration [13–15]. We therefore analyzed cdc42 expression and activation in these ACTN4 knockdown cells. Western blotting of whole cell lysates indicated that the expression level of Cdc42 was the same in A549-luc-C8-KD-ACTN4s and A549-luc-C8-cont cells (Figure 3F). However the activation of Cdc42 in A549-luc-C8-KD-ACTN4s was significantly decreased in comparison with that in A549-luc-C8-cont cells (Figure 3G).

### Chemosensitivity of the cells to cytotoxic drugs used for ADJ

**Figure 4:**
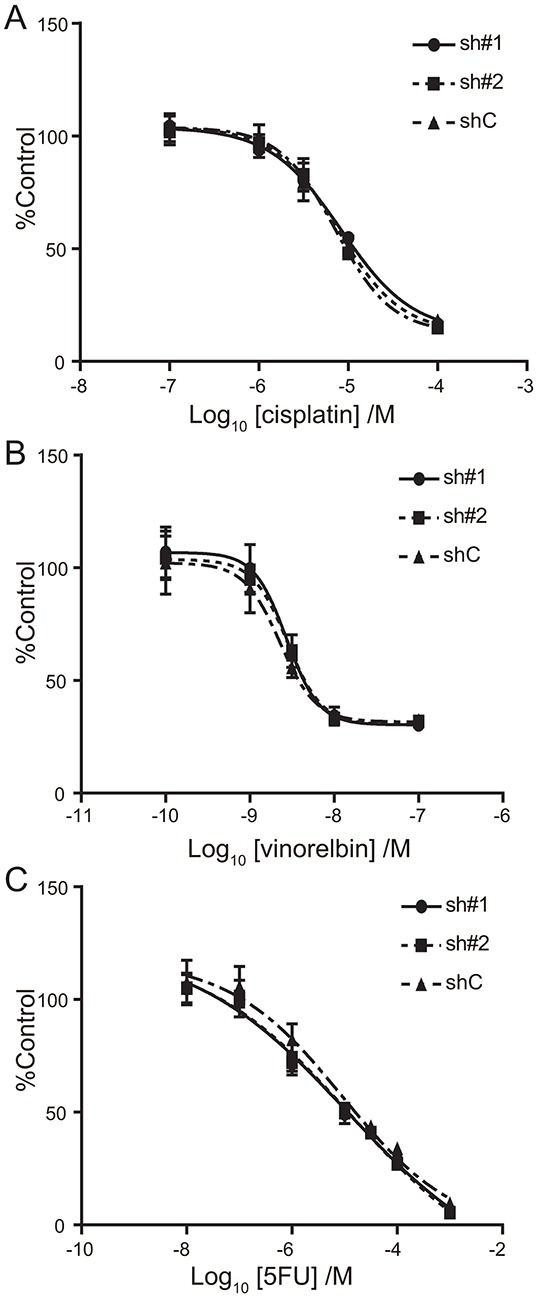
Effect of ACTN4 knockdown using shRNA of ACTN4 on chemosensitivity to cytotoxic drugs IC50 curves for cisplatin **A.** vinorelbin **B.** and 5-FU **C.** for A549 cells in which ACTN4 or control shRNAs were transfected (circles; sh#1, squares sh#2, and triangles shC).

The chemosensitivity of A549-luc-C8-cont and A549-luc-C8-KD-ACTN4 cells to cytotoxic drugs (cisplatin, vinorelbin, and 5-fluorouracil (5-FU)) was examined using dose dependent proliferation assays. No significant differences in chemosensitivity to cisplatin, vinorelbin or 5-FU were found between A549-luc-C8-cont and A549-luc-C8-KD-ACTN4 cells (Figure 4A–4C).

### Evaluation of the involvement of ACTN4 in A549 metastatic ability by using an animal transplantation model

**Figure 5:**
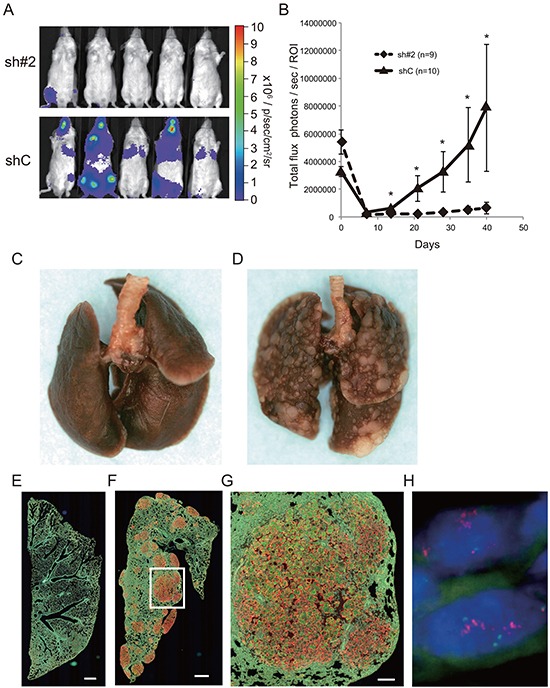
Effect of ACTN4 knockdown using shRNA of ACTN4 on cell metastatic ability in an in vivo animal model **A, B.** Evaluation of luciferase signals from the injected mice using an in vivo imaging system (IVIS). (A) Representative photographs of luciferase signals from mice injected with the different A549 cells (40 days after injection). (B) Alterations in luciferase signal intensities over time after cell injection (diamonds; sh#2, and triangles shC. Bars; SEM, and *P < 0.01 Mann Whitney t-test). **C, D.** Representative murine lungs on day 40 (C, sh#2; D, shC). **E–G.** Immunohistochemical analysis of murine lung on day 40 with anti-ACTN4 (red) and anti-cytokeratin 19 (green) antibodies (E, sh#2; F, G; shC). Protein overexpression of ACTN4 was detected in the metastatic lesions of murine lung. Bar in E and F indicates 500 μm, and that in G indicates 100 μm. **H.** Representative fluorescence in situ hybridization analysis of ACTN4 in the metastatic region of animal models.

We next examined the effect of ACTN4 knockdown on cell metastatic ability by using an animal transplantation model in which A549-luc-C8-KD-ACTN4 (sh#2) or A549-luc-C8-cont (shC) was injected into the tail vain of SCID mice. On day 0, fluorescent signals were detected in the lungs of SCID mice that were injected with either A549-luc-C8-KD-ACTN4 or A549-luc-C8-cont. Seven days after injection (day 7), fluorescent signals were not detected in the lungs of any of these mice. Although there was an increase in signal intensity in the lungs of mice that were injected with A549-luc-C8-cont starting from day 14, no increase in signal intensity was detected in the lungs of mice that were injected with A549-luc-C8-KD-ACTN4 over the 40 days after injection. The signal intensity in the lungs of A549-luc-C8-cont-injected mice compared with that of A549-luc-C8-KD-ACTN4-injected mice was significantly higher from day 14 until day 40 after injection (Figure 5A (40 days after injection) and 5B). Macro- and microscopic examination of the lungs of these mice indicated significantly greater metastatic lesions of the lung in the A549-luc-C8-cont injected group in comparison with the A549-luc-C8-KD-ACTN4 injected group (Figure 5C–5G). Gene amplification of ACTN4 was observed in these lung metastatic lesions by fluorescence in situ hybridization (FISH) analysis (Figure 5H). These data suggested that ACTN4 contributed to lung metastasis of A549 cells injected in vivo.

## DISCUSSION

This is the first report that expression and gene amplification of ACTN4 have the potential to be a predictive biomarker for ADJ of early stage NSCLC. Zhu et al. examined the comprehensive mRNA expression profile of patients who were enrolled by JBR.10, and subsequently identified a gene signature of 15 genes that could accurately predict prognosis and the survival benefits of ADJ [5]. This gene signature obtained using microarray data could not only predict the prognosis for disease specific survival of the patients who were observed without ADJ in JBR.10 (HR 15.02, 95% CI 5.12 – 44.04), but it could also predict the clinical benefit of cisplatin based ADJ (HR 0.33, 95% CI 0.17 – 0.63) in the patients with a high risk signature. In contrast, in the patients with a low risk signature, the risk for disease specific death in ADJ significantly increased in comparison with the observation group (HR 3.67 95% CI 1.22 – 11.06). That study suggested that a gene signature of 15 genes is a predictive biomarker for the efficacy of cisplatin based ADJ [5].

In general, ACTN4 is a prognostic biomarker for several kinds of cancers. ACTN4 has also been strongly associated with cancer metastatic ability [9]. Moreover gene amplification of ACTN4 is a prognostic biomarker for stage-I lung adenocarcinoma patients who never underwent ADJ after surgery [10]. A prognostic biomarker for patients with stage-I who have never undergone ADJ means that such a biomarker has the potential to be a predictive biomarker for efficacy of ADJ of these patients. Thus, the logic behind the therapeutic strategy is that the patients who may have micrometastatic lesions should undergo ADJ. In the present reanalysis of JBR.10, we demonstrated that a significant clinical benefit of cisplatin based ADJ for overall survival was recognized only in the ACTN4 (+) subgroup (HR 0.273, 95% CI 0.079 – 0.952) (Figure 1C). This clinical benefit was never seen in the subgroups of baseline (Figure 1A) or of ACTN4 (−) (Figure 1D). As stated above, our previous study demonstrated that gene amplification of ACTN4 was significant prognostic biomarker for the stage-I patients with lung adenocarcinoma who never underwent adjuvant chemotherapy. In the present study, the patients of baseline consisted not only of the subgroup of ACTN4 (+), but also included the ACTN4 (−) subgroup. Whereas the subgroup of ACTN4 (+) could get clinical benefit with ADJ, which extended the overall survival times in comparison with OBS, in contrast, the subgroup of ACTN4 (−) never got a survival benefit with ADJ. Therefore, when the patients of baseline were considered, no statistical significance in ACTN4 as prognostic biomarker was recognized between the subgroup of ACTN4 (+) and ACTN4 (−) (Figure 1B).

Those data suggested that ACTN4 was not a simple prognostic factor for stage-I/IIA patients with NSCLC. Moreover, in another study, using immunohistochemical analysis (IHC) we recently retrospectively demonstrated that stage-II/IIIA patients with lung adenocarcinoma in which the ACTN4 protein is overexpressed got clinical benefit for overall survival from ADJ with cisplatin and vinorelbin. However patients without overexpression of ACTN4 never got clinical benefit from ADJ (Shiraish et al, in submission). These retrospective IHC data are consistent with the JBR.10 data of this study. Moreover, our previous data demonstrated that some patients who strongly overexpressed the ACTN4 protein also had significant gene amplification of ACTN4, including patients with lung adenocarcinoma, salivary gland carcinoma, pancreatic cancer, and ovarian cancer [12, 16–18]. Therefore one of the mechanisms for protein overexpression of ACTN4 is amplification of the *ACTN4* gene.

In vitro and animal experiments suggested that the invasive phenotype of A549, which has gene amplification of ACTN4, could be reduced by knock-down of ACTN4. This phenomenon could be explained by the fact that ACTN4 is involved in the formation of the cellular processes that are associated with cancer invasion, but is not involved in cell proliferation or in adhesion to endothelial cells. Based on those data, we concluded that gene amplification and protein overexpression of ACTN4 are potential biomarkers for evaluation of the metastatic ability of early stage NSCLC. The chemosensitivity of the cells was not changed by infection of shRNA of ACTN4. These data suggested that ACTN4 is a possible predictive biomarker for the selection of patients who should undergo ADJ, i.e., the selection of patients who have the risk of potential micro metastatic lesions beyond the limit of resected areas. Thus ACTN4 is not a biomarker for specific selection of the most effective cytotoxic drug, such as cisplatin and vinorelbin. We think that ACTN4 is a potential biomarker for choosing patients who should undergo ADJ, regardless of the cytotoxic drug. Our retrospective data suggested that patients in the subgroup of ACTN4 (+) with stage-I lung adenocarcinoma who underwent ADJ with tegafur-uracil displayed improved overall survival (data not shown). These clinical and preclinical data do not contradict the possibility that ACTN4 is a predictive biomarker of the efficacy for ADJ for early stage NSCLC, which is based on the use of ACTN4 measurements to evaluate the metastatic ability of each individual patient.

One limitation of this study is that this study was not a prospective study for the demonstration of the clinical benefit of ACTN4 for ADJ. Our cut-off value of the expression level of ACTN4 mRNA was calculated from the database of JBR.10 using bioinformatics and it has not yet been confirmed by other methods. Moreover, although we previously showed that gene amplification of ACTN4 is a prognostic biomarker of stage-I lung adenocarcinoma, in the present study we could not confirm the status of the copy number of ACTN4 from this database. In addition, this study was a reanalysis of stage-I/II NSCLC, but not of stage-I of adenocarcinoma. We are currently considering the clinical application of ACTN4 measurement by using IHC or FISH with a specific antibody or fluorescent DNA probe, respectively, for analysis of ACTN4 levels in surgical specimens of early stage NSCLC.

In order to determine the clinical applicability of ACTN4 as a predictive biomarker in stage-IB, the copy number of ACTN4 should be evaluated using IHC and FISH from clinical samples that were prospectively enrolled by clinical trials.

## MATERIALS AND METHODS

### Reanalysis of the public database of JBR.10

Snap-frozen tumor tissues that were enrolled by the National Cancer Institute Canadian Clinical Trial Group JBR.10 were collected prospectively. The mRNA was extracted from these snap-frozen tumor tissues and transcriptomic profiles were examined using the U133A oligonucleotide microarray by Zhu et al. [5]. The microarray data have been enrolled at the National Center for Biotechnology Information Gene Expression Omnibus (GSE14814). Information of clinical data, survival times, and mRNA expression profiles have been described in this database by the National Cancer Institute Canadian Clinical Trial Group. We downloaded these data, and then reanalyzed the impact of ACTN4 on the efficacy of ADJ in JBR.10 using this database.

### Statistical analysis and bioinformatics

The microarray data and clinical information of patients were downloaded from GEO (GSE14814). The 5-year overall survivals determined by Kaplan-Meier methods and Cox regression analysis were analyzed by the R-project (http://www.r-project.org/) [19].

### Lung adenocarcinoma cell lines, antibodies and drugs

A549 was purchased from the American Type Culture Collection (Manassas, VA). PC3, PC9, and PC14 were purchased from Immuno-Biological Laboratories (Takasaki, Japan). RERF-LC-KJ, and RERF-LC-MS were purchased from the Human Science Research Resources Bank (Osaka, Japan). A549-luc-C8 was purchased from Caliper Life Sciences (Hopkinton, MA). Anti-actinin-4 rabbit polyclonal antibody (Ab-2) and mouse monoclonal antibody (13G9, Abnova, Taiwan) were produced as described previously [12, 20, 21]. Anti-β-actin mouse monoclonal antibody (AC-15), anti-Cdc42 mouse monoclonal antibody (M152) and anti-cytokeratin 19 rabbit polyclonal antibody (ab53119) were purchased from Abcam (Cambridge, UK). Cisplatin (Nippon Kayaku, Japan), vinorelbin (Kyowa Hakko Kirin, Japan) and 5-fluorouracil (5FU, Wako Pure Chemical Industries, Japan) were purchased as indicated.

### Absolute quantification of ACTN4

The separated and eluted peptides were detected using QTRAP5500 in the setting of multiplexed multiple reaction monitoring (MRM) transitions as shown in Figure 2C. All mass spectrometry (MS) analysis was performed with the positive ion mode with spray voltage at 2000 V. Other MS settings are shown below: Curtain gas (CUR), Collision gas, Ion source gas, Declustering potential (DP) and Entrance potential (EP) were 25, 6, 15, 80 and 13, respectively. Collision Cell Exit Potential was set at 6 or 13 for light or heavy amino acids. A standard curve was generated by using the solution equally mixed with serial dilutions of the light peptide (0, 25, 50, 100, 200 fmol/μL) and the internal standard heavy peptide (50 fmol/μL). The quantitation value was calculated from the peak area ratio of each transition for the light peptide to that for the heavy peptide, and the amount of peptide was calculated as the average of the 4 quantification values determined from the 4 sets of transitions [22].

### Immunoblotting

Protein samples were fractionated by sodium dodecyl sulfate-polyacrylamide gel electrophoresis (SDS-PAGE) and blotted onto Immobilon-P membranes (Millipore, Billerica, MA). After incubation with primary antibodies at 4°C overnight, signals were detected with relevant horseradish peroxidase-conjugated anti-mouse or anti-rabbit IgG antibody and ECL Western Blotting Detection Reagents (GE Healthcare, Giles, UK), according to the manufacturer's protocol [20].

### Immunofluorescence cytochemistry

Cells grown on poly-L-lysine-coated cover glasses (BD Biosciences, San Jose, CA) and were fixed with 4% paraformaldehyde for 10 minutes at room temperature. The cells were incubated with anti-actinin-4 mouse monoclonal antibody and then with anti-mouse IgG Alexa Fluor 488 (Life Technologies). Filamentous actin fibers were visualized with Alexa Fluor 594 phalloidin (Life Technologies), as described previously [8, 20].

### RNA interference (RNAi)

Silencer negative control RNA and siRNA targeting ACTN4 (Hs_ACTN_5 and Hs_ACTN4_10) (Qiagen) were used at a final concentration of 50 nM. Transient transfection was performed using Lipofectamine 2000 (Life Technologies) in accordance with the manufacturer's protocol.

Stable knockdown was performed using the Mission shRNA lentiviral transduction system (Sigma-Aldrich, St. Louise, MO). Two shRNA constructs targeting ACTN4 (TRCN0000055784 and TRCN0000055785) and two control constructs (empty vector control SHC001V) were selected from the RNAi Consortium shRNA library. A549-luc-C8 cells were plated in 100 μL RPMI 1640 medium per well on 96-well plates and incubated overnight. The medium was then changed to growth medium containing hexadimethrine bromide (Sigma) at a concentration of 8 μg/mL to enhance transduction efficiency. After 16 h incubation with lentiviral particles, cells were selected with puromycin (Sigma) at a concentration of 10 μg/mL.

### Transfection

ACTN4 cDNA was amplified by PCR using TaKaRa Ex Taq HS DNA polymerase (TaKaRa Bio Inc., Kyoto, Japan) and the primers: ACCATGGACTACAAGGACGACGATGACAAGATGGTGGACTACCACGCGG and TCATCTATCTAGATCTTCACAGGTCGCTCTCGCCATAC. The PCR product was cloned into the pcDNA 3.1/V5-His TOPO TA vector (Life Technologies). A549 cells plated at a density of 3 × 10^5^ cells in six-well plates were transfected with 2.5 μg of the ACTN4-pcDNA construct or control plasmid using Lipofectamine 2000 (Life Technologies) in accordance with the manufacturer's protocol.

### Cell migration assay

Cells (1 × 10^5^) were seeded into each insert of a 24-well Biocoat Matrigel Invasion Chamber (BD Biosciences Discovery Labware, Franklin Lakes, NJ) in triplicate. Fetal bovine serum was added to a final concentration of 10% in the lower chambers. After incubation for 22 h, cells that had migrated into the other sides of the membranes were stained with Diff-Quik kit (Sysmex Corp., Kobe, Japan). The total number of migrated cells in 5 microscopic fields was counted and averaged.

### Wound healing assay

Cells were grown to confluency on glass coverslips, and a “wound” was then introduced into the monolayers with a plastic pipette tip. After incubation for 24 h, the cells were photographed [7].

### Cell proliferation assay

Cells were plated at a density of 2 × 10^3^ cells/well into 96-well tissue culture clusters in sextuplicate, and cell viability was measured with the CellTiter-Glo Luminescent Cell Viability Assay (Promega, Madison, WI) using a GloMax 96 Microplate Luminometer (Promega, Madison, WI). The value of ATP activity for each cell at 0 h was set as 1.

### Scanning electron microscopy

Cells grown on poly-L-lysine-coated cover glasses were fixed with 2% glutaraldehyde at 4°C overnight. The cells were then dehydrated in an ascending ethanol series. After critical-point drying using liquid carbon dioxide the specimens were coated with platinum and examined using a scanning electron microscope (JSM-7500; JEOL Ltd., Tokyo, Japan) [20].

### Cell adhesion assays

Cell adhesion assays were carried out using an endothelial cell adhesion assay kit (Millipore, ECM645) according to the manufacturer's instructions. HUVEC (5 × 10^4^ cells/mL) were seeded on 96-well plates at a density of 5 × 10^3^ cells/well and were cultured for 2 days. The HUVEC monolayer was treated with 10 ng/mL TNFα for 4 h. A549-luc-C8 cells were added (2 × 10^5^ cells/well) to the HUVEC monolayers and incubated for 30 min. The plate was washed extensively and the adherent A549-luc-C8 cells were measured with the Steady-Glo Luciferase Assay System (Promega, Madison, WI) using a GloMax 96 Microplate Luminometer (Promega). Assays were run in triplicate on plates. Cell adherence was calculated as the ratio of the luciferase activity of the adhered A549-luc-C8, where the luciferase activity in the added A549-luc-C8 was taken as 1.

### G-LISA Cdc42 activation assay

Activation of Cdc42 was also followed using G-LISA activation assay kits from Cytoskeleton according to the manufacturer's instructions. Assays were run in triplicate. The signal was measured at 490 nm, using a microplate spectrophotometer.

### Cytotoxic assay

Cells were plated at 2 × 10^3^ cells/well into 96-well tissue culture clusters in sextuplicate. After 24 hours, cultured cells were treated with a series of different concentrations of cytotoxic reagents. After 48 hours, cell viability was measured with the CellTiter-Glo Luminescent Cell Viability Assay (Promega, Madison, WI) using GloMax 96 Microplate Luminometer (Promega, Madison, WI). Data analysis was done with GraphPad Prism 6.05 for Windows (GraphPad Software, La Jolla Californina USA).

### Animal experiment

Male severe combined immunodeficiency (SCID) mice (C.B-17/Icr-scid) were purchased from Clea Japan (Tokyo, Japan) and were maintained in a specific pathogen-free environment. A549-luc-C8 cells (5 × 10^6^ cells) were injected into tail veins. For the detection of bioluminescence emission, mice were anesthetized with 2% inhaled isoflurane (Mylan, Osaka, Japan), and D-luciferin (Promega, Madison, WI) was injected intraperitoneally at a dose of 150 mg/Kg. Assessment of photonic emissions (photons s-1) was performed 10 min after the injection using the In Vivo Imaging System (IVIS) 100 (Xenogen, Alameda, CA). All animal experimental procedures were reviewed and approved by the ethics committee of the National Cancer Center Research Institute (Tokyo, Japan).

### Immunohistochemistry (IHC)

Immunohistochemical analysis was performed as described previously [23]. Mouse tissue sections were stained with anti-actinin-4 monoclonal antibody and anti-cytokeratin 19 rabbit polyclonal antibody. The second antibodies were anti-mouse IgG Alexa Fluor594 and anti-rabbit IgG Alexa Flour488 (Life Technologies).

## SUPPLEMENTARY FIGURE


