# Supplementary material for: Efficacy of adjuvant chemotherapy for non-small cell lung cancer assessed by metastatic potential associated with ACTN4
Source: Oncotarget. 2016 Apr 21;7(22):33165–78. doi: 10.18632/oncotarget.8890 (PMC5078083; doi:10.18632/oncotarget.8890)
Supplement: Supplementary file 1 [file oncotarget-07-33165-s001.pdf]

SUPPLEMENTARY FIGURE

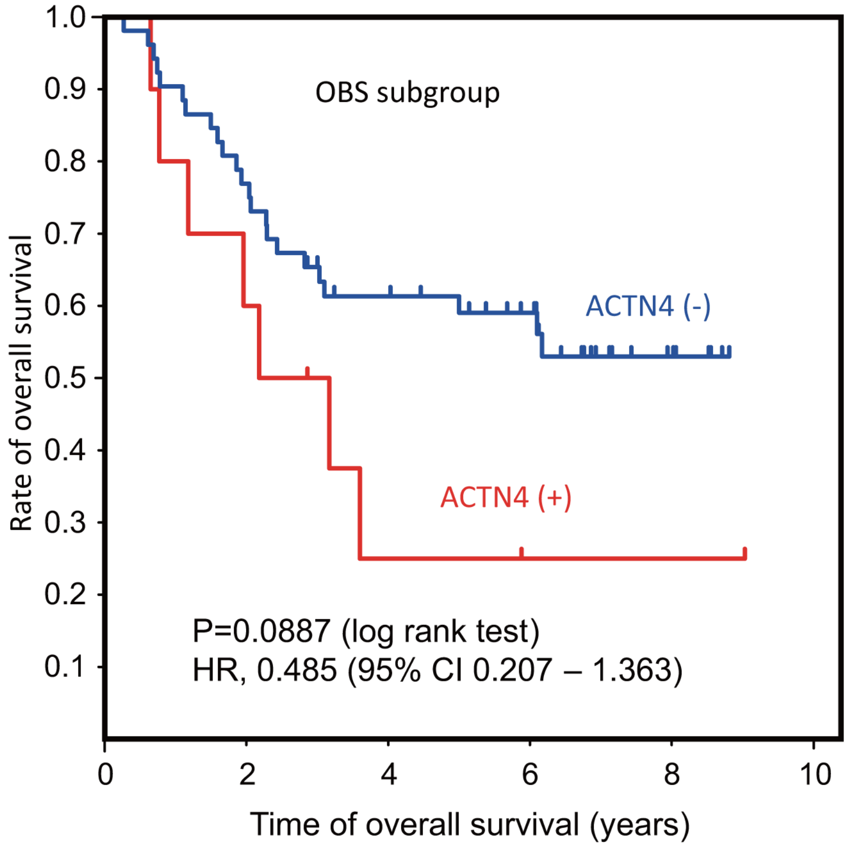

Supplementary Figure S1: Kaplan Meier curves of overall survival were constructed for OBS subgroup. ACTN4 (-) (bule line) and ACTN4 (+) (red line).
